# The associations between social comparison on social media and young adults’ mental health

**DOI:** 10.3389/fpsyg.2025.1597241

**Published:** 2025-08-08

**Authors:** Justine Le Blanc-Brillon, Jean-Simon Fortin, Livia Lafrance, Sébastien Hétu

**Affiliations:** ^1^Laboratory NECS, Department of Psychology, Université de Montréal, Montreal, QC, Canada; ^2^Centre Interdisciplinaire de Recherche sur le Cerveau et l’Apprentissage, Montreal, QC, Canada

**Keywords:** social media, psychology, mental health, self-esteem, depression, social psychology

## Abstract

**Introduction:**

Social networking sites (SNSs) have become an integral part of daily life, raising concerns about their potential impact on mental health. One key mechanism through which SNSs may affect wellbeing is social comparison. The present research aimed to examine the mediating role of social comparisons (upward and downward) in the relationship between SNSs use and self-esteem (global and physical).

**Methods:**

Study 1 (*N* = 139; female 51%; 73% White), conducted during the COVID-19 pandemic, tested whether perceived exposure to social comparisons mediated the relationship between Instagram use and self-esteem.

**Results:**

Results revealed that upward comparisons mediated the association between Instagram use and lower global self-esteem, but no significant mediation was found for physical self-esteem. Study 2 (*N* = 413; 58% female; 62% White), conducted post-pandemic, extended these findings by including two SNSs (Instagram and Facebook), the extremity of upward comparisons (how far superior the comparison target is perceived to be), social feedback (responses or evaluations from others) and measures of depressive symptoms. As expected, exposure to upward comparisons negatively mediated the relationship between SNSs use and self-esteem (both global and physical), and positively mediated the relationship between SNSs use and depressive symptoms. However, contrary to our hypothesis, frequent SNSs users engaged in less extreme upward comparisons, partially buffering the negative impact of extreme upward comparisons.

**Discussion:**

Together, these findings highlight the crucial role of both exposure to and extremity of upward social comparisons in the complex relationship between SNSs use and mental health. These two factors contribute significantly though modestly to the effects of SNSs on self-esteem and depressive symptoms (*R*^2^ between 6 and 9%), underscoring the need for further research on individual and contextual variables that may mitigate their adverse psychological consequences.

## 1 Introduction

Social networking sites (SNSs) continue to grow in popularity, playing an increasingly central role in users’ lives (Pew Research Center, 2024; Villanti et al., 2017). This trend is concerning, as a growing body of research points to a connection between SNSs use and declines in various aspects of mental health and quality of life (e.g., Cunningham et al., 2021; Purba et al., 2023; Vannucci et al., 2020). One mental health outcome frequently linked to SNSs use is self-esteem, a psychological construct that is fundamental to wellbeing (Mann et al., 2004). The suspected effect of SNSs use on self-esteem may stem from the myriad opportunities for upward social comparisons-comparing oneself to someone perceived as superior-these platforms offer to users (Appel et al., 2016). Given the importance of this societal issue, there is a pressing need for more empirical data clarifying the role of social comparisons in the relationship between SNSs use and self-esteem.

SNSs are web-based platforms that enable users to (1) create a public or semi-public profile within a defined network, (2) establish a list of connections with other users, and (3) interact with their own and others’ connections on the platform (Boyd and Ellison, 2007). Users engage with SNSs for various purposes, including maintaining interpersonal relationships, relaxing, and expressing opinions (Whiting and Williams, 2013). Importantly, users also report using SNSs both to share glimpses of their lives and to keep track of what others are doing (Whiting and Williams, 2013). Beyond these uses, some studies suggest that SNSs can foster social support, enhance a sense of belonging, and provide opportunities for self-expression and identity exploration, which may positively influence users’ wellbeing (Manago et al., 2012; Nabi et al., 2013).

Over the last decade or so, SNSs have become increasingly integrated into users’ daily lives (Pew Research Center, 2024; Villanti et al., 2017). A recent survey reported that 72% of Americans use social media, with usage rising to 84% among 18–29-year-olds (Pew Research Center, 2021). Similarly, 77.6% of Canadians are active on at least one SNSs, with this figure increasing to 90% among those aged 35 and younger (Gouvernement du Canada, 2021). Instagram now boasts over 2.3 billion active users (Ruby, 2023), Facebook has surpassed 2.9 billion (Baron, 2023), and TikTok, launched in 2016, has already reached over 1 billion monthly active users (Seignol de Swarte, 2022). This steady rise in SNSs use is a matter of concern. While the effects of SNSs use are unlikely to be entirely negative (e.g., Smith et al., 2021; Spottswood and Wohn, 2020), a substantial body of research suggests a strong association with poorer mental health outcomes. Meta-analyses of correlational studies consistently indicate a positive relationship between SNSs use and depressive symptoms (Cunningham et al., 2021; Ivie et al., 2020; Liu et al., 2022; Shannon et al., 2022). Furthermore, increased time spent on SNSs has been linked to heightened feelings of loneliness (Liu and Baumeister, 2016; Zhang et al., 2022), a greater incidence of eating disorders (Holland and Tiggemann, 2016; Sharma and Vidal, 2023), and lower life satisfaction (Huang, 2017). Notably, SNSs use is also associated with reduced self-esteem (Liu and Baumeister, 2016; Saiphoo et al., 2020). This is especially concerning, as self-esteem is often considered a key determinant of mental health, potentially affecting a broad spectrum of mental health outcomes (Mann et al., 2004).

Self-esteem refers to an individual’s overall positive or negative attitude toward themselves (Rosenberg et al., 1995). Researchers commonly distinguish between global and domain-specific self-esteem. Global self-esteem reflects a person’s general sense of self-worth, while domain-specific self-esteem pertains to self-assessments in particular areas, such as physical appearance or academic achievement (Donnellan et al., 2011). In the context of SNSs, where appearance is often highlighted (Popat and Tarrant, 2023), physical self-esteem appears particularly vulnerable. Indeed, on these platforms, individuals frequently present an idealized version of themselves, sharing polished and highly curated images (Manago et al., 2008; Young et al., 2022).

Both global and physical self-esteem appear to play important roles in mental health and wellbeing. Meta-analytic evidence of correlational studies indicates medium-sized correlations between global self-esteem and both life satisfaction (Diener and Diener, 1995) and wellbeing (Muris and Otgaar, 2023). Longitudinal studies have further demonstrated that self-esteem serves as a predictor of depressive symptoms (Sowislo and Orth, 2013), eating disorders (Colmsee et al., 2021), suicidal behavior (Soto-Sanz et al., 2019), self-harm (Junker et al., 2019), and peer victimization (van Geel et al., 2018). Additionally, higher levels of self-esteem have been reported to predict academic achievement (Valentine et al., 2004) and the quality of social relationships (Harris and Orth, 2020). Correlational studies also suggest that global self-esteem is negatively associated with loneliness (Mahon et al., 2006), substance abuse (Martínez-Casanova et al., 2024), and risky sexual behavior (Ahn and Yang, 2023). As for physical self-esteem, related constructs such as body image and body appreciation have been the focus of extensive research. A meta-analysis by Linardon et al. (2022) found that body appreciation is positively correlated with wellbeing and negatively correlated with depression and eating disorders. Additionally, body image has been reported to be negatively associated with self-harm (Oktan, 2017), as well as suicidal ideation and behavior (Angelakis et al., 2016).

Given the importance of self-esteem for mental health, the rise in SNSs use over the last decade is concerning. Indeed, a substantial body of evidence demonstrates that SNSs use is negatively associated with both global (Andreassen et al., 2017; Liu and Baumeister, 2016; Saiphoo et al., 2020) and physical self-esteem (Fardouly and Vartanian, 2016; Saiphoo et al., 2020).

According to social comparisons theory (Festinger, 1954), humans possess a fundamental drive to assess their opinions and abilities. Individuals use social comparisons as a mechanism to enhance their self-understanding by evaluating themselves in relation to others (Festinger, 1954). Similarly, ranking theory posits that social comparisons is an evolutionarily ancient process allowing individuals to assess their position in the social hierarchy (Gilbert, 1992). Social comparisons can take three primary forms: downward, lateral, and upward comparisons (Guyer and Vaughan-Johnston, 2020). Downward comparisons occur when individuals evaluate themselves against others they perceive as inferior on a given dimension, while lateral comparisons involve evaluations against those perceived as similar or equal. Upward comparisons, by contrast, involve assessing oneself against others perceived as superior.

The highly curated and idealized content prevalent on SNSs encourages users to engage in upward social comparisons, where they compare themselves to seemingly superior others (Henriques and Patnaik, 2020). Studies have consistently shown that frequent use of SNSs is linked to an increase in these upward comparisons (Gomez et al., 2022; Schmuck et al., 2019; Vogel et al., 2014). Drawing on social comparison theory, these upward comparisons can be expected to negatively affect self-perceptions, particularly self-esteem. From the perspective of ranking theory, such comparisons may also threaten one’s perceived social standing, thereby increasing psychological vulnerability. Together, these theories inform the central hypothesis of the present research: that upward comparisons mediate the relationship between SNSs use and reduced self-esteem.

Evidence suggests that upward comparisons can negatively affect both global and physical self-esteem. Research on this relationship includes studies that examine direct effects, showing that upward comparisons independently harm self-esteem (e.g., Ho et al., 2016; Rodgers et al., 2015; McComb and Mills, 2022; McComb et al., 2023), as well as studies exploring mediation pathways, where upward comparisons act as a mediator between other variables, such as social media use and self-esteem (e.g., Wang et al., 2017; Schmuck et al., 2019; Vogel et al., 2014). This effect is well-documented across a range of research designs, including correlational (Ho et al., 2016; Wang et al., 2017), longitudinal (Rodgers et al., 2015; Schmuck et al., 2019), and experimental studies (McComb and Mills, 2022; McComb et al., 2023; Vogel et al., 2014). The consistent findings across these methodologies highlight the robust nature of this effect on both forms of self-esteem. Given that upward social comparisons are positively associated with SNSs use and linked to lower self-esteem, it may be a key mechanism mediating the association between higher SNSs use and reduced self-esteem—a hypothesis supported by current research.

Indeed, several studies have examined the mediating role of social comparisons in the relationship between SNSs use and *global* self-esteem (e.g., Midgley et al., 2021; Vogel et al., 2014; Wang et al., 2017). For instance, Vogel et al. (2014) found that the negative impact of Facebook use on self-esteem is mediated by the tendency to engage in upward social comparisons on SNSs, while downward comparisons had no significant effect. Similarly, Midgley et al. (2021) exposed one group to a SNSs context and another to a non-social media context. Participants in the SNSs condition reported lower self-esteem by the end of the study compared to those in the non-SNSs condition. Their findings indicated that this relationship between exposure to SNSs and self-esteem was mediated by an increased likelihood of making upward comparisons, consistent with the results of Vogel et al. (2014).

Other studies have investigated the mediating role of social comparisons on SNSs in the relationship between SNSs use and *physical* self-esteem (e.g., Scully et al., 2020; Tiggemann and Zaccardo, 2015; Wang et al., 2017). Overall, these studies indicate that upward social comparisons mediate the relationship between SNSs use and physical self-esteem. For example, Scully et al. (2020) found, in their correlational study, that the tendency to engage in upward comparisons on SNSs mediates the negative relationship between social media use and body satisfaction. Similarly, Tiggemann and Zaccardo (2015), in an experimental study, assigned participants to one of two groups: one exposed to ‘fitspiration’ images on social media, designed to evoke body-related upward comparisons, and the other exposed to travel images. Those exposed to fitspiration images reported lower appearance-based self-esteem after the task, with upward appearance-based comparisons mediating the negative relationship between exposure to fitspiration content and self-esteem.

Upward social comparisons and its suspected negative effects on self-esteem may vary based on both context and individual characteristics. Key questions remain: under what conditions do these comparisons lead to detrimental outcomes, and how do these processes unfold? While research in this area is still emerging, several factors have been proposed that might influence the relationship between SNSs use, upward comparisons, and both global and physical self-esteem. Variables such as the extremity—defined as the perceived gap between the individual and the comparison target—of social comparisons, the specific SNSs platform, social feedback (i.e., responses or evaluations from others), and gender all seem to play a role. For instance, Midgley et al. (2021) suggest that extreme upward comparisons, where the comparison targets are perceived as significantly superior, can negatively mediate the relationship between SNSs use and self-esteem. Nonetheless, research on the extremity of social comparisons in the context of SNSs use and its impact on mental health remains limited. Similarly, the potential differences in mental health effects and underlying mechanisms across platforms remain largely underexplored, despite their likely significance. Limniou et al. (2022) compared Facebook and Instagram and found that Instagram users exhibited more problematic usage patterns, suggesting that Instagram may foster more addictive behaviors. Because Instagram emphasizes visual content like photos and videos, some researchers suggest that it may influence self-esteem and wellbeing differently than platforms such as Facebook (Faelens et al., 2021). Social feedback is another factor gaining attention in the context of SNSs and mental health. Positive social feedback (e.g., receiving more likes) has been associated with enhanced self-esteem (Burrow and Rainone, 2017; Marengo et al., 2021). However, conflicting evidence suggests that this same feedback may also be linked to reduced self-esteem (Limniou et al., 2022). Lastly, research focusing on adolescence reveals that social media’s impact on mental health tends to be more pronounced in females (Barthorpe et al., 2020; Kelly et al., 2019; Twenge et al., 2022).

Initial studies examining the impact of SNSs largely emphasized a straightforward, linear connection between SNSs use and wellbeing, overlooking the more intricate dynamics at play (Kross et al., 2021). It has become increasingly clear, however, that the influence of SNSs varies based on the context, with outcomes ranging from positive to harmful (Cingel et al., 2022; Krause et al., 2019). While there has been a growing interest in uncovering the mechanisms that link SNSs usage to mental health outcomes (Meier and Johnson, 2022; Verduyn et al., 2020), this field of research is still in its infancy. One major gap in the current literature is the limited understanding of the specific mechanisms through which SNSs exert their influence on mental wellbeing (Kross et al., 2021). Moreover, although an increasing amount of data suggests the mediating role of upward social comparisons in the relationship between media usage and both global (e.g., Midgley et al., 2021; Vogel et al., 2014; Wang et al., 2017) and physical self-esteem (e.g., Scully et al., 2020; Tiggemann and Zaccardo, 2015; Wang et al., 2017; Vogel et al., 2014), no research has investigated this role for both types of self-esteem in the same participants. Examining global and physical self-esteem at the same time allows for a more nuanced understanding of how social comparisons impact distinct domains of self-concept. Theoretically, this approach acknowledges that self-esteem is a multidimensional construct, with different domains potentially responding differently to upward comparisons. Methodologically, it ensures within-subject comparability, thereby reducing confounds and enabling more precise modeling of individual differences. Investigating these two concepts simultaneously would then allow for a better understanding of which aspect(s) of self-esteem is/are more affected by the use of these platforms. Lastly, very few studies in this field focus on multiple SNSs simultaneously. For example, the majority of studies either focus solely on Facebook (Gonzales and Hancock, 2011; Tazghini and Siedlecki, 2013; Vogel et al., 2014) or on Instagram (Dion, 2016; Hill and Denman, 2016; Paramboukis et al., 2016). However, the nature of the content found on each of these applications differs greatly. Instagram is primarily image-based and often promotes idealized self-presentation, while Facebook includes more textual content, mixed media, and interactions grounded in existing offline relationships (Sheldon and Bryant, 2016). Additionally, Instagram tends to be more popular among younger users and is associated with appearance-focused comparison, whereas Facebook has a broader age demographic and is linked to different social comparison processes (Huang, 2017). Therefore, the consequences of these different SNSs may not be the same from one application to another, as this meta-analysis has shown (Akkaş and Turan, 2024). Investigating two different SNSs would thus better distinguish the individual impacts of each of these platforms and can serve as a basis for issuing more tailored recommendations.

Therefore, to fill these gaps in the literature, we conducted two studies designed to enhance our understanding of the mechanisms through which social media use affects mental health. By examining how upward social comparisons impact different domains of self-esteem and symptoms of depression across multiple platforms, these studies offer insights that may inform the development of safer, more mindful social media practices, as well as future digital literacy and mental health interventions. In study 1, which took place during the COVID-19 pandemic, we examined the mediating role of exposure to social comparisons (upward and downward) in the relationship between SNSs usage and self-esteem (both global and physical). In study 2, which was pre-registered, we examined the mediating role of perceived exposure to social comparisons (upward and downward) in the relationship between SNSs usage and (i) self-esteem (global, physical, and academic) and (ii) symptoms of depression. We also investigated the role of other variables: the extremity of social comparisons, the specific SNSs platform (Instagram versus Facebook), social feedback (its valence and quantity), and gender. This study has been approved by the Ethics Committee CEREP (Comité d’éthique de la recherche en éducation et en psychologie) of the Université de Montréal (CEREP-21-081-D).

## 2 Study 1

### 2.1 Method

#### 2.1.1 Hypothesis

*H1:* SNSs will be related to lower self-esteem (physical and global) and this relation will be partially mediated by the exposure to upward social comparisons—but not downward comparisons—with more exposure to upward comparisons resulting in lower self-esteem (global and physical).

This online study was not preregistered. However, all statistical models that were tested are transparently reported in the present manuscript.

#### 2.1.2 Participants

139 American or Canadian (71 female) participants aged between 18 and 35 years old (M_age_ = 28.13, SD = 4.16) were recruited on the Amazon Mechanical Turk (MTurk) platform in October 2021, during the COVID-19 pandemic,^1^ and directed to the online questionnaire on Limesurvey. The inclusion criteria were: (i) being aged between 18 and 35 years, (ii) being a Canadian or American citizen, (iii) currently being active on Instagram, and (iv) being fluent in English. Participants who did not respond correctly to the attention check question (*N* = 16) were excluded from the analyses.

#### 2.1.3 Measures

Participants completed a series of questionnaires administered in a random order.

##### 2.1.3.1 Instagram usage

This self-reported questionnaire was adapted from the Facebook usage questionnaire in Vogel et al. (2014) and consists of 5 items (Cronbach’s alpha (*α*) = 0.87): the frequency of usage, the frequency of posting on Instagram (photos and stories) and actions taken on Instagram (likes and comments)—to be answered using a seven-point Likert scale ranging from 1 (never) to 7 (multiple times a day). The summed score, ranging from 5 (very low usage) to 35 (very high usage), was used as a measure of Instagram usage.

##### 2.1.3.2 Perceived exposure to social comparisons

To assess participants’ perceived exposure to upward and downward social comparisons, we used the Social comparisons questionnaire created by Vogel et al. (2014). Participants had to evaluate to what extent they focused on people better and worse than themselves on Instagram (1 = not at all to 5 = a great deal) with two questions: one for the exposure to upward comparisons and one for the exposure to downward comparisons.

##### 2.1.3.3 Global self-esteem

Participants completed the Rosenberg Self-Esteem scale (*α* = 0.81) (Rosenberg, 1965; Ruddell, 2020). They indicated their agreement with 10 statements on a four-point Likert scale (1 = Strongly Disagree to 4 = Strongly Agree). The overall score on the Rosenberg self-esteem scale is calculated by summing all items and interpreted using a scale ranging from 10 (Poor self-esteem) to 40 (Excellent self-esteem). The overall score was used as a measure of global self-esteem.

##### 2.1.3.4 Physical self-esteem

Participants completed the Body-Esteem scale (*α* = 0.90) (Franzoi and Shields, 1984; Cecil and Stanley, 1997). They indicated their feelings toward 35 statements on a five-point Likert scale (1 = Have strong negative feelings to 5 = Have strong positive feelings). The overall score on the Body-Esteem scale is calculated by summing all items and interpreted using a scale ranging from 35 (Poor physical self-esteem) to 175 (Excellent physical self-esteem).

##### 2.1.3.5 Sociodemographic questionnaire

Participants had to answer a sociodemographic questionnaire to collect information such as their age, sex, gender, and socioeconomical status.

#### 2.1.4 Statistical analysis

First, correlations were calculated between all key variables: amount of Instagram use, self-esteem (physical, global), perceived exposure to social comparisons-perception of the frequency or intensity of comparison with others-(upward and downward). Additionally potential covariables (age and socioeconomical status) were explored. Then, to determine whether the relationship between Instagram use and self-esteem (physical, global) was mediated by the exposure to upward and/or downward social comparisons, mediation analyses using unstandardized regression coefficients were conducted. We used the method described by Preacher and Hayes (2008) and the PROCESS macro (Model 4; Preacher and Hayes, 2008), with 5,000 bootstrap samples to estimate indirect effects. Models included a direct relationship between Instagram use and self-esteem (physical, global), as well as a parallel mediation through upward and downward social comparisons (total of 2 models). Models controlling for covariables identified in the exploratory analyses were also tested and gender was tested as a moderator of the direct link and the link between mediators and our dependent variables. No significant moderation effects were found.

### 2.2 Results

Table 1 presents the sociodemographic characteristics of the final sample. Normality tests conducted on each variable confirmed that all distributions were normal.

**Table 1:** Sociodemographic characteristics of the final sample in Study 1.

Sociodemographic characteristics of participants	*n*	%
Gender		
Female	71	51.1
Male	68	48.9
Other	0	
Ethnicity		
White	101	72.7
African	11	7.9
Asian	13	9.4
Latino	5	3.8
Native American	7	5.0
Other	2	1.4
Main occupation		
Full-time work	117	84.1
Part-time work	9	6.5
Full-time study	5	3.6
Unemployed	6	4.3
Other	2	1.4
Civil status		
Married	74	53.2
Common law spouse	1	0.7
Divorced	1	0.7
Separated	0	0
Single	56	40.3
Unmarried couple	7	5.0

Table 2 displays the correlations among the key variables. The frequency of Instagram use was positively correlated with both global self-esteem, (*r*(139) = 0.18, *p* = 0.04), and physical self-esteem, (*r*(139) = 0.38, *p* < 0.01). Frequency of Instagram use was also associated with higher perceived exposure to both upward (*r*(139) = 0.57, *p* < 0.01) and downward (*r*(139) = 0.51, *p* < 0.01) social comparisons on Instagram as well as gender (*r*(139) = −0.19, *p* = 0.03). Results for potential covariables can be found in Supplementary Table 1.

**Table 2:** Correlations among the key dependent measures in Study 1.

Key dependent variables	Instagram use	Global self-esteem	Physical self-esteem	Upward comparison	Downward comparison	Age	Gender
Instagram use	–	0.18*	0.38**	0.57**	0.51**	−0.03	−0.19*
Global self-esteem	–		0.46**	−0.09	−0.12	0.24**	0.05
Physical self-esteem	–	–	–	0.14	0.30*	0.11	0.01
Upward comparison	–	–	–	–	0.46**	−0.03	−0.22**
Downward comparison	–	–	–	–	–	−0.08	−0.15
Age	–	–	–	–	–	–	0.49**
Gender	–	–	–	–	–	–	–

Significance level: *<0.05; **<0.01.

#### 2.2.1 Global self-esteem mediation model

**Figure 1:**
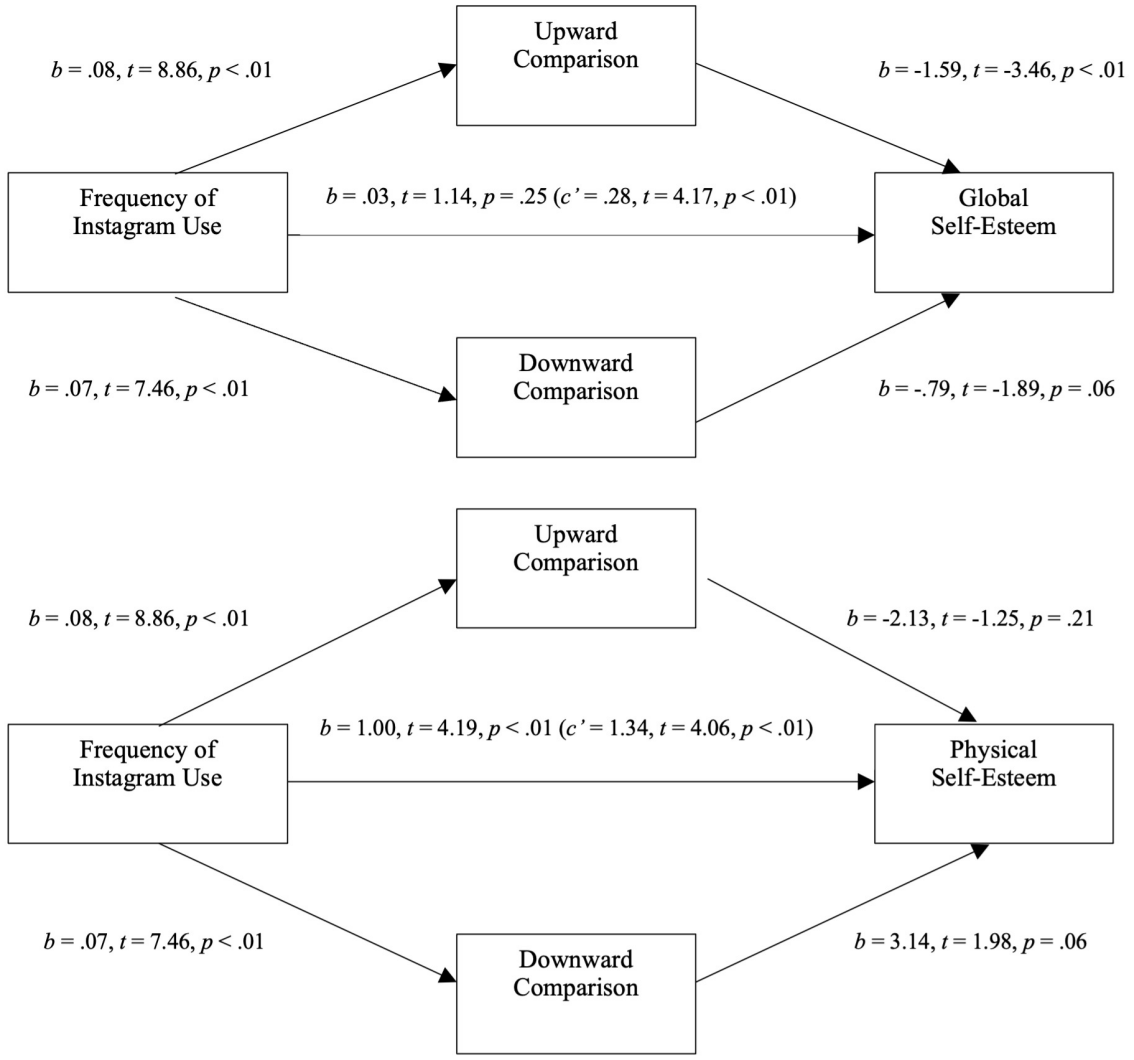
Mediation analysis for study 1. Top: Mediation analysis between the frequency of Facebook use and global self-esteem through the exposure to social comparison (upward and downward). Bottom: Mediation analysis between the frequency of Facebook use and physical self-esteem through the extremity of social comparison (upward and downward).

As shown in Figure 1, when considering the mediators, frequency of Instagram use was a significant positive predictor of global self-esteem (*b* = 0.28, *t* = 4.17, *p* < 0.01), indicating that, contrary to our hypothesis, participants with higher Instagram use had better global self-esteem. Instagram use was also a positive predictor of both perceived exposure to upward (*b* = 0.08, *t* = 8.86, *p* < 0.01) and downward (*b* = 0.07, *t* = 7.46, *p* < 0.01) social comparisons, but only upward comparisons was significantly negatively associated with global self-esteem. Only the mediation path through upward was significant (*b* = −0.13; 95%, CI [−0.22, −0.04]). These results suggest that while Instagram use seems to be associated to higher global self-esteem, this effect is reverse when considering the mediating role of upward comparisons: the more people use Instagram, the more they are exposed to upward social comparisons which leads to lower global self-esteem. This model explained 8% (*p* < 0.01) of the variance of global self-esteem.

#### 2.2.2 Physical self-esteem mediation model

As shown in Figure 1, when considering the mediators, frequency of Instagram use was a significant positive predictor of physical self-esteem (*b* = 1.05, *t* = 4.24, *p* < 0.01), indicating that, contrary to our hypothesis, participants with higher Instagram use had a better perception of their bodies. The mediation paths through perceived exposure to upward comparisons (*b* = −0.17; 95%, CI [−0.60, 0.13]) and downward comparisons (*b* = 0.22; 95%, CI [−0.02, 0.51]) were not significant.

### 2.3 Discussion

Study 1 aimed to examine whether perceived exposure to social comparisons mediates the relationship between Instagram use and young adults’ global and physical self-esteem. Contrary to our initial hypothesis—and much of the prior literature suggesting a detrimental effect—the results revealed that frequency of Instagram use was a significant positive predictor of both global and physical self-esteem. Previous research has often highlighted the risks of upward social comparison on SNSs, showing that increased use is associated with reduced self-esteem (Schmuck et al., 2019; Sherlock and Wagstaff, 2019). However, findings across studies have not always been consistent. Recent work suggests that the relationship between SNSs use and wellbeing is highly context-dependent and may vary based on user motivations, content type, and platform engagement styles (Verduyn et al., 2020; Meier and Johnson, 2022; Akkaş and Turan, 2024). It is increasingly evident that the effects of SNSs are context-dependent—sometimes beneficial and sometimes detrimental (Cingel et al., 2022; Krause et al., 2019).

Despite the unexpected direction of the overall association, as hypothesized, perceived exposure to upward comparisons was a negative predictor of global self-esteem and mediation analyses suggested that the relationship between Instagram use and lower global self-esteem partially operates through an increase in upward social comparisons. This finding is in line with previous evidence that social comparisons on social networking sites negatively influence self-esteem (McComb et al., 2023; Schmuck et al., 2019; Wang et al., 2017). In contrast, social comparisons did not mediate the relationship between Instagram use and physical self-esteem, contradicting our hypothesis and previous research (Prieler et al., 2021; Scully et al., 2020).

One possible explanation lies in the nature of the measure used. The Body-Esteem Scale (Franzoi and Shields, 1984) assesses satisfaction with specific body parts (e.g., feet, arms), which may not reflect the appearance-focused comparisons prevalent on Instagram. As such, this scale may be less sensitive to the kinds of upward comparisons users are exposed to. Future research may benefit from using broader or more appearance-focused measures of body image, such as the Body Esteem Scale for Adolescents and Adults (Mendelson et al., 2001) or the Body Appreciation Scale (Avalos et al., 2005).

Several contextual factors related to the COVID-19 pandemic may also help explain why Instagram use was positively associated with self-esteem in this study. For instance, Cauberghe et al. (2021) found that individuals experiencing higher levels of loneliness were more likely to turn to SNSs as a way to cope with the absence of social interaction. For many, platforms like Instagram may have helped users maintain social connections during lockdowns, which could have increased feelings of social belonging—a known contributor to self-esteem (Denissen et al., 2008; Leary and Baumeister, 2000). Additionally, it is likely that individuals engaged more in active SNSs use rather than passive scrolling during lockdowns, which may have shifted the negative relationship between SNSs use and self-esteem commonly reported in pre-pandemic studies (Wallinheimo and Evans, 2021). Indeed, it has been proposed that passive SNSs use—where individuals consume content without interacting with others—can be damaging to wellbeing, while active use—where individuals interact with others—has a neutral or even beneficial effect (Verduyn et al., 2021). However, that these interpretations remain speculative. We did not collect data on variables such as loneliness, type of SNS use (active vs. passive), or social feedback, which limits our ability to test these explanations empirically. Finally, while our findings diverge from the dominant narrative, other studies suggest that the relationship between SNSs use and mental health remained negative during the pandemic (Draženović et al., 2023; Haddad et al., 2021; Marciano et al., 2022). Our unexpected finding of a non-significant link between perceived exposure to upward comparisons and physical self-esteem could also be related to the shift in the nature of content shared on SNSs during the COVID-19 pandemic. With people being isolated and confined to their homes, the content typically conducive to upward social comparisons—such as gym or travel photos—diminished significantly.

Other limitations of this study should be acknowledged. First, several variables that could have impacted the results were not included in our study such as the valence and quantity of social feedback received on SNSs. Substantial positive social feedback (e.g., likes, comments) may boost users’ self-esteem, while abundant negative feedback (e.g., negative comments, insults, etc.) may harm it. Additionally, we did not gather data on the extremity of social comparisons. According to (Midgley et al., 2021), engaging in more extreme upward social comparisons—where the gap between the user and the comparison target is larger—can have a more pronounced effect on users’ self-esteem than less extreme comparisons. Finally, participants were recruited via MTurk, a method known to introduce potential demographic biases, such as higher digital literacy and specific cultural or political orientations, which may affect generalizability of our findings (Chandler et al., 2019).

## 3 Study 2

### 3.1 Method

To address some of study 1 limitations and provide new data about the role of social comparison in the impacts of SNSs usage, we ran a second study. This study was conducted on two SNSs (Instagram and Facebook), collected data after the COVID-19 pandemic, included several new measures related to social feedback and comparison extremity, used a new measure of physical self-esteem, included a control dependent variable (academic self-esteem), and explored the effects on depressive symptoms. This study was preregistered (Le Blanc-Brillon and Hétu, 2023).

#### 3.1.1 Hypotheses

*H2a (pre-registered):* The exposure to upward social comparisons—but not downward comparisons—will mediate the relationship between SNSs use and self-esteem (physical and global, but not academic), with more exposure to upward comparisons resulting in lower self-esteem (global and physical, but not academic).

*H2b (pre-registered):* The extremity of upward social comparisons—but not downward comparisons—will mediate the relationship between SNSs use and self-esteem (physical and global, but not academic), with more extreme upward comparisons leading to a greater decrease in self-esteem (global and physical, but not academic).

*H2c (exploratory):* The exposure to upward social comparisons—but not downward comparisons—will mediate the relationship between SNSs use and depressive symptoms, with more exposure to upward comparisons leading to increased depressive symptoms.

*H2d (exploratory):* The extremity of upward social comparisons—but not downward comparisons—will mediate the relationship between SNSs use and depressive symptoms, with more extreme upward comparisons leading to increased depressive symptoms.

*H2e (pre-registered):* These effects (decreased self-esteem and increased depressive symptoms) were expected to be more pronounced in the Instagram condition than in the Facebook condition, due to the more visually focused and appearance-based content on Instagram.

*H2f (exploratory):* These effects (decreased self-esteem and increased depressive symptoms) were expected to be moderated by variables such as the quantity and valence of social feedback received on social media and participants’ gender.

#### 3.1.2 Participants

A total of 413 American or Canadian participants were recruited on the Amazon Mechanical Turk platform in June 2023 and redirected on an online Limesurvey survey. They were randomly assigned to the Instagram (*n* = 206; 118 females) or the Facebook conditions (*n* = 207; 122 females). All the participants were aged between 18 and 35 years old (M_ageInstagram_ = 28.53, SD = 3.89; M_ageFacebook_ = 28.54, SD = 3.93). The inclusion criteria were the same as in the Study 1, except that participants had to be currently active on both Facebook and Instagram. Participants who did not respond correctly to the attention check question (*N*_Facebook_ = 6; *N_Instagram_* = 9) or who provided an unrelated response to the open-ended question in the social media usage questionnaire (*N_Facebook_* = 2) (“Did you ever take a break from the Instagram application? If yes, why?”) were excluded from the analyses.

#### 3.1.3 Measures

Participants completed a series of questionnaires administered in a random order.

##### 3.1.3.1 SNSs usage

The same questionnaire as Study 1 was used to assess participants’ social media use but the wording was adapted to the social media site, either Facebook or Instagram, depending on the condition.

##### 3.1.3.2 Perceived exposure to social comparisons

The social comparisons questionnaire used in Study 1 was used to assess participants’ exposure to upward and downward comparisons with the wording adapted depending on the condition.

##### 3.1.3.3 Perceived extremity of social comparisons

Participants completed an adapted version of the MacArthur Scale of Subjective Social Status (Adler et al., 2000). They rated their own social rank and the one of the profiles they follow on a scale from 1 (bottom of the hierarchy) to 10 (top of the hierarchy). Perceived extremity of social comparison was calculated by subtracting the social rank of the profiles from their own social rank and taking the absolute value. If participants had a higher rank than the profiles, this value was entered as the extremity of downward social comparisons (with 0 as the value for extremity of upward social comparisons). If participants had a lower rank than the profiles, this value was entered as the extremity of upward social comparisons (with 0 as the value for extremity of upward social comparisons).

##### 3.1.3.4 Quantity and valance of social feedback on SNSs

Participants completed a custom questionnaire consisting of two items. One to assess the valence of the feedback received on Facebook or Instagram (depending on the condition), answered using a 7-point Likert scale ranging from 1 (very negative) to 7 (very positive). One to evaluate the quantity of social feedback received on Facebook or Instagram (depending on the condition), answered using a 7-point Likert scale ranging from 1 (none at all) to 7 (a lot).

##### 3.1.3.5 Global self-esteem

As in study 1, the Rosenberg self-esteem scale (*α* = 0.81) was used to assess participants’ global self-esteem.

##### 3.1.3.6 Physical self-esteem

Participants completed the Body-esteem scale for adolescents and adults (*α* = 0.90) (Mendelson et al., 2001), a self-reported questionnaire consisting of 23 items aimed at evaluating the participant’s appreciation of their physical appearance. Participants answered using a four-point Likert scale ranging from “Strongly agree” to “Strongly disagree.” The total score is calculated by summing the score of all items and interpreted using a scale ranging from 23 (poor body esteem) to 92 (excellent body esteem).

##### 3.1.3.7 Academic self-esteem

Participants completed the Academic Self-Esteem Questionnaire (*α* = 0.80) (Duclos, 1996) which consists of 9 items answered using a 5-point Likert scale ranging from “Strongly disagree” to “Strongly agree.” Scores can be interpreted using a scale ranging from 9 (poor academic self-esteem) to 45 (excellent academic self-esteem). Since school-related of self-esteem should not be affected by social media usage it was used as a control variable.

##### 3.1.3.8 Depression

Participants completed the Beck Depression Inventory (*α* = 0.84) (Beck et al., 1987), a self-reported questionnaire consisting of 21 items aimed at assessing the severity of depressive symptoms present in the participants during the last 2 weeks. They responded using a Likert scale ranging from 0 (least severe) to 3 (most severe). The score is calculated by summing all items and interpreted using a scale ranging from 0–13 (minimal) to 29–63 (severe).

##### 3.1.3.9 Sociodemographic questionnaire

The same sociodemographic questionnaire as in Study 1 was used.

#### 3.1.4 Statistical analysis

First, exploratory correlations were conducted between all key variables: amount of social media use, self-esteem (physical, global, academic), depression, perceived exposure to-perception of the frequency or intensity of comparison with others-and extremity of social comparisons-how extreme or large the gap is perceived to be between oneself and comparison targets-(upward and downward), and potential covariables. Then, to determine whether the relationship between social media use and self-esteem (physical, global, and academic) was mediated by the exposure to upward and/or downward social comparisons and/or the extremity of these comparisons, mediations were conducted using the method described by Preacher and Hayes (2008) and the PROCESS macro [Model 4, 15; (Preacher and Hayes, 2008)], with 5,000 bootstrap samples to estimate indirect effects. All models included a direct relationship between social media use (Instagram or Facebook) and self-esteem (physical, global, and academic) or depression, as well as a parallel mediation through upward and downward social comparisons (perceived exposure and extremity). The models controlling for covariables identified in the exploratory analysis and inclusion of social feedback (valence and quantity) and gender as moderators of the direct link, the link between our independent variable and mediators, and the link between mediators and our dependent variables were also tested but only reported if significant. These analyses were conducted separately for Facebook and Instagram, and for physical, global, academic self-esteem, and depression (total of 8 models).

### 3.2 Results

Results presented in the following sections come from our preregistered analyses (Le Blanc-Brillon and Hétu, 2023).

Table 3 presents the sociodemographic characteristics of the final sample. Normality tests conducted on each variable confirmed that all distributions were normal. Table 4 presents the average scores for key variables and comparison between the Facebook and Instagram samples. Overall, samples were not statistically different for perceived exposure to upward and downward social comparisons, global and scholar self-esteem and depression scores. Social media usage, physical self-esteem and perceived extremity of upward comparisons were slightly higher in the Instagram sample, whereas perceived extremity of downward comparisons was slightly lower in the Instagram sample.

**Table 3:** Sociodemographic characteristics of the final sample in Study 2.

Sociodemographic characteristics of participants	Facebook	Instagram
	*n*	*n*
Gender		
Female	122	118
Male	80	79
Other	5	9
Ethnicity		
White	127	130
African	28	36
Asian	24	18
Latino	23	15
Native American	3	3
Other	2	4
Education		
High school	47	49
Technical school	9	5
Undergraduate studies	81	82
Graduate studies	44	39
Postgraduate studies	24	29
Other	2	2

**Table 4:** Average scores for key variables and differences between samples in Study 2.

Key variables in study 2	Facebook	Instagram	t-test
	M (SD)	M (SD)	*t* (*p*)
Age	28.54 (3.93)	28.35 (3.89)	0.47 (0.97)
Utilization	26.67 (15.68)	30.4 (15.10)	−1.72 (<0.01)
Upward comparison	2.97 (1.31)	3.23 (1.26)	−2.07 (0.42)
Downward comparison	2.33 (1.06)	2.16 (1.10)	1.63 (0.77)
Global self-esteem	27.37 (6.06)	27.45 (6.12)	−0.14 (0.86)
Physical self-esteem	56.61 (14.04)	59.32 (11.93)	−2.11 (0.03)
BDI	14.08 (12.78)	13.75 (13.20)	0.26 (0.48)
Scholar self-esteem	26.14 (4.91)	26.34 (4.91)	−0.36 (0.99)
Social feedback	12.38 (5.39)	12.28 (5.41)	0.20 (0.54)
Extremity upward	1.13 (1.40)	1.68 (1.76)	−3.54 (<0.01)
Extremity downward	0.36 (0.93)	0.20 (0.60)	2.12 (<0.01)

Tables 5, 6 show the correlations among the key dependent measures. Both Instagram and Facebook use were not significantly correlated with global or physical self-esteem (*p* > 0.05). However, for both platforms, frequency of use was positively correlated with both upward (Instagram: *r*(206) = 0.16, *p* = 0.02; Facebook: *r*(207) = 0.28, *p* < 0.001) and downward (Instagram: *r*(206) = 0.18, *p* = 0.01; Facebook: *r*(207) = 0.19, *p* < 0.01) social comparisons. Surprisingly, more frequent use of both platforms was associated with lower perceptions of upward comparison extremity (Instagram: *r*(206) = −0.15, *p* = 0.03; Facebook: *r*(207) = −0.16, *p* = 0.03), suggesting that individuals who reported more frequent use of both platforms tended to perceive upward comparisons as less extreme. For both platforms, identifying as female (coded as 1 in the analyses) was significantly correlated with lower global self-esteem (Instagram: *r*(206) = −0.19, *p* < 0.01; Facebook: *r*(207) = −0.26, *p* < 0.01), lower physical self-esteem (Instagram: *r*(206) = −0.32, *p* = < 0.01; Facebook: *r*(207) = −0.26, *p* < 0.01) and higher depression scores (Instagram: *r*(206) = 0.23, *p* = < 0.01; Facebook: *r*(207) = 0.23, *p* < 0.01). Results for potential covariables can be found in Supplementary Tables 2, 3.

**Table 5:** Correlations among the key dependent measures in Study 2—Facebook condition.

Key dependent variables	Facebook use	Global self-esteem	Physical self-esteem	Depression	Upward comparison	Downward comparison	Extremity of upward comparison	Extremity of downward comparison	Academic self-esteem	Age	Gender
Facebook use	–	−0.01	0.08	0.11	0.28**	0.19**	−0.16*	0.13	−0.01	0.07	0.08
Global self-esteem	–	–	0.68**	−0.67**	−0.28**	−0.10	−0.24**	0.12	0.50**	0.12	−0.26**
Physical self-esteem	–	–	–	−0.57**	−0.13	−0.02	−0.22**	0.10	0.25**	−0.06	−0.26**
Depression	–	–	–	–	0.29**	0.14*	0.22**	−0.01	−0.30**	0.00	0.23**
Upward comparison	–	–	–	–	–	0.36**	0.05	−0.30	−0.16*	0.02	0.12
Downward comparison	–	–	–	–	–	–	−0.25**	0.19**	−0.09	0.11	−0.01
Extremity upward comparison	–	–	–	–	–	–	–	−0.32**	−0.12	−0.02	0.05
Extremity downward Comparison	–	–	–	–	–	–	–	–	0.03	0.02	0.04
Academic self-esteem	–	–	–	–	–	–	–	–	–	−0.01	−0.04
Age	–	–	–	–	–	–	–	–	–	–	−0.01
Gender	–	–	–	–	–	–	–	–	–	–	–

Significance level: *<0.05; **<0.01.

**Table 6:** Correlations among the key dependent measures in Study 2—Instagram condition.

Key dependent variables	Instagram use	Global self-esteem	Physical self-esteem	Depression	Upward comparison	Downward comparison	Extremity of upward comparison	Extremity of downward comparison	Academic self-esteem	Age	Gender
Instagram use	–	−0.06	0.02	0.08	0.16*	0.18*	−0.15*	0.04	−0.13	−0.14*	−0.08
Global self-esteem	–	–	0.64**	−0.64**	−0.24**	−0.17*	−0.27**	0.03	0.51**	0.16*	−0.19**
Physical self-esteem	–	–	–	−0.48**	−0.29**	−0.06	−0.27**	0.02	0.30**	0.04	−0.32**
Depression	–	–	–	–	0.19**	0.16*	0.25**	−0.00	−0.39**	−0.03	0.23**
Upward comparison	–	–	–	–	–	0.33**	0.18*	0.05	−0.12	0.00	0.10
Downward comparison	–	–	–	–	–	–	−0.21**	0.16*	−0.15	−0.03	0.01
Extremity upward comparison	–	–	–	–	–	–	–	−0.32**	−0.19*	−0.05	0.25**
Extremity downward Comparison	–	–	–	–	–	–	–	–	−0.07	−0.07	−0.02
Academic self-esteem	–	–	–	–	–	–	–	–	–	−0.09	−0.06
Age	–	–	–	–	–	–	–	–	–	–	0.03
Gender	–	–	–	–	–	–	–	–	–	–	–

#### 3.2.1 Facebook condition

**Figure 2:**
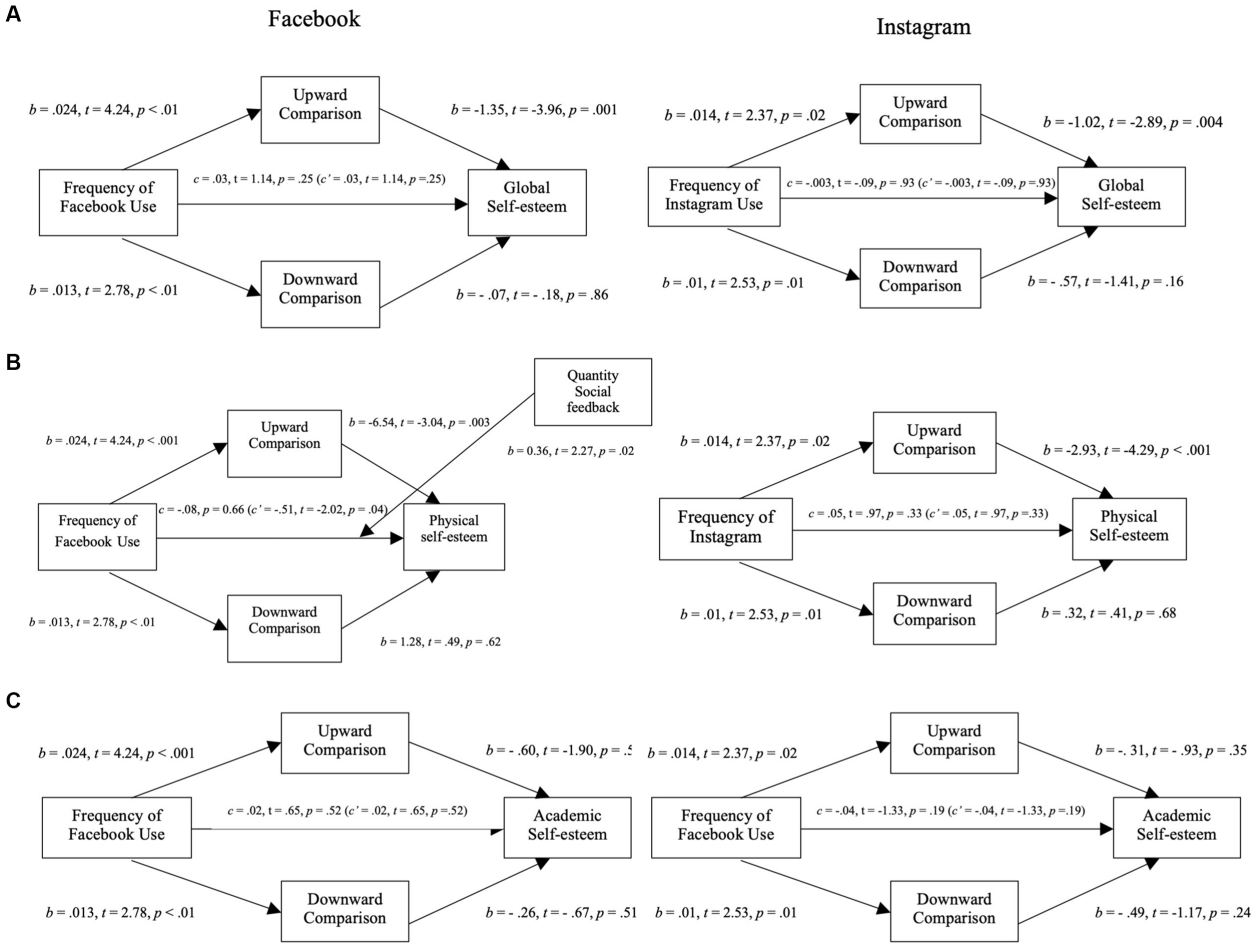
Mediation analysis for self-esteem variables through exposure to social comparison in Study 2. **(A)** Mediation analysis between the frequency of Facebook use (left)/Instagram use (right) and global self-esteem through the exposure to social comparison (upward and downward). **(B)** Mediation analysis between the frequency of Facebook use (left)/Instagram use (right) and physical self-esteem through the exposure to social comparison (upward and downward). **(C)** Mediation analysis between the frequency of Facebook use (left)/Instagram use (right) and academic self-esteem through the exposure to social comparison (upward and downward).

**Figure 3:**
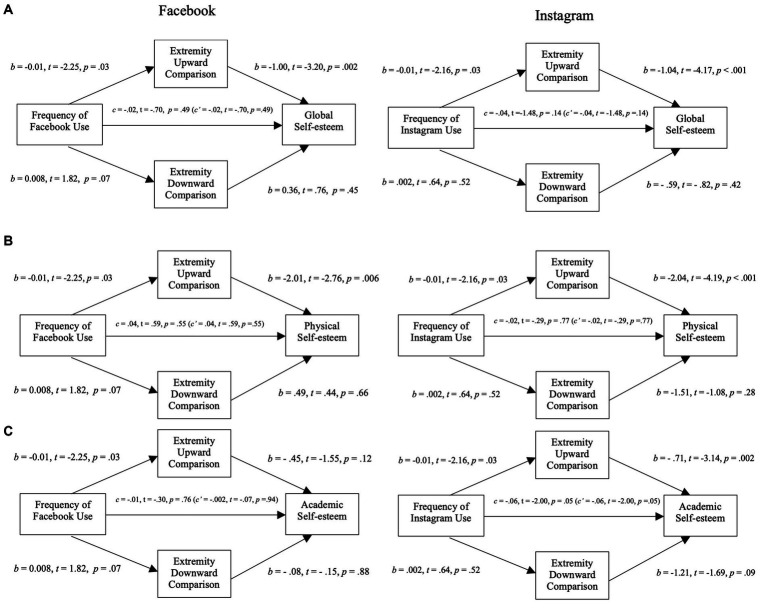
Mediation analysis for self-esteem variables through extremity of social comparison in Study 2. **(A)** Mediation analysis between the frequency of Facebook use (left)/Instagram use (right) and global self-esteem through the extremity of social comparison (upward and downward). **(B)** Mediation analysis between the frequency of Facebook use (left)/Instagram use (right) and physical self-esteem through the extremity of social comparison (upward and downward). **(C)** Mediation analysis between the frequency of Facebook use (left)/Instagram use (right) and academic self-esteem through the extremity of social comparison (upward and downward).

The following section reports the effects of Facebook use on our preregistered dependent variables, focusing on testing the mediating role of perceived exposure to and extremity of social comparisons. The left side of Figures 2, 3 show the models with perceived exposure (2) to and extremity of (3) upward and downward social comparisons as mediators.

##### 3.2.1.1 Global self-esteem

The negative relation between Facebook usage and global self-esteem was fully mediated by perceived exposure to upward social comparisons (*b* = −0.03; 95% CI [−0.06, −0.01]): more Facebook usage was associated with higher perceived exposure to upward social comparisons (*b* = 0.024, *t* = 4.24, *p* < 0.0001) which was negatively related to global self-esteem (*b* = −1.35, *t* = −3.96, *p* = 0.0001). This model explained 8% (*p* < 0.01) of the variance of global self-esteem and was not improved by the addition of moderators or covariates.

Contrary to our hypothesis we found a fully mediated positive relation between Facebook usage and global self-esteem with extremity of social comparisons as the mediators (*b* = 0.01; 95% CI [0.002, 0.03]). More specifically, while we found that higher perceived extremity of upward social comparisons was negatively associated with reduced global self-esteem (*b* = −1.00, *t* = −3.20, *p* = 0.002), contrary to our hypothesis, Facebook usage was associated with lower perceived extremity (*b* = −0.01, *t* = −2.25, *p* = 0.03). This model explained 6% (*p* < 0.01) of the variance of global self-esteem and was not improved by the addition of various moderators or covariates.

##### 3.2.1.2 Physical self-esteem

The negative relation between Facebook usage and physical self-esteem was partially mediated by perceived exposure to upward social comparisons (*b* = −0.04; 95% CI [−0.09, 0.002]): more Facebook usage was associated with higher perceived exposure to upward social comparisons which was negatively related to physical self-esteem (*b* = −6.54, *t* = −3.04, *p* = 0.002). When controlling for mediators, higher Facebook usage was negatively related to physical self-esteem (*c’* = −0.51 *p* = 0.04).

Contrary to our hypothesis, we found a fully mediated positive relation between Facebook usage and physical self-esteem with extremity of social comparisons as the mediators (*b* = 0.02; 95% CI [0.002, 0.07]). More specifically, while we found that perceived extremity of upward social comparisons was negatively related with physical self-esteem (*b* = −2.01, *t* = −2.76, *p* = 0.006), contrary to our hypothesis, more Facebook usage was associated with lower perceived extremity of upward social comparisons.

In both models, the inclusion of quantity of social feedback as a moderator revealed a significant interaction with Facebook usage on physical self-esteem (*b* = 0.36, *t* = 2.27, *p* = 0.02), suggesting that that the relationship between Facebook usage and physical self-esteem depends on the quantity of social feedback received. Specifically, higher Facebook usage was associated with better physical self-esteem among individuals who received a greater amount of social feedback. These moderated mediation models explained 8% (*p* < 0.01) of the variance in physical self-esteem when perceived exposure to social comparisons was the mediator and 5% (*p* = 0.03) when perceived extremity of social comparisons was the mediator.

##### 3.2.1.3 Academic self-esteem

In models assessing academic self-esteem (Figures 2, 3), Facebook usage was not directly nor indirectly related to academic self-esteem. Neither exposure to nor extremity of upward or downward comparisons were significantly associated with academic self-esteem (*p* > 0.05 for both b).

#### 3.2.2 Instagram condition

The following section reports the effects of Instagram use on our preregistered dependent variables, focusing on the exposure to and extremity of social comparisons as potential mediators. The right side of Figures 2, 3 show the models with perceived exposure (2) to and extremity of (3) upward and downward social comparisons as mediators.

##### 3.2.2.1 Global self-esteem

The negative relation between Instagram usage and global self-esteem was fully mediated by perceived exposure to upward social comparisons (*b* = − 0.02; 95% CI [−0.04, −0.002]): more Instagram usage was associated with higher perceived exposure to upward social comparisons (*b* = 0.014, *t* = 2.37, *p* = 0.02) which was negatively related to global self-esteem (*b* = −1.02, *t* = −2.89, *p* = 0.004). This model explained 7% (*p* < 0.01) of the variance of global self-esteem and was not improved by the addition of moderators or covariates.

Contrary to our hypothesis we found a fully mediated positive relation between Instagram usage and global self-esteem with extremity of social comparisons as the mediators (*b* = 0.01; 95% CI [0.002, 0.04]). More specifically, while we found that higher perceived extremity of upward social comparisons was negatively associated with reduced global self-esteem (*b* = −1.04, *t* = −4.217, *p* < 0.001), contrary to our hypothesis, Instagram usage was associated with lower perceived extremity (*b* = −0.01, *t* = −2.16, *p* = 0.03). This model explained 8% (*p* < 0.01) of the variance of global self-esteem and was not improved by the addition of various moderators or covariates.

##### 3.2.2.2 Physical self-esteem

The negative relation between Instagram usage and physical self-esteem was fully mediated by perceived exposure to upward social comparisons (*b* = −0.04; 95% CI [−0.09, −0.01]): more Instagram usage was associated with higher perceived exposure to upward social comparisons which was negatively related to physical self-esteem (*b* = −2.93, *t* = −4.29, *p* < 0.001). This model explained 9% (*p* < 0.01) of the variance of physical self-esteem and was not improved by the addition of moderators or covariates.

Contrary to our hypothesis, we found a fully mediated positive relation between Instagram usage and physical self-esteem with extremity of social comparisons as the mediators (*b* = 0.02; 95% CI [0.01, 0.07]). More specifically, while we found that perceived extremity of upward social comparisons was negatively related with physical self-esteem (*b* = −2.04, *t* = −4.19, *p* < 0.001), contrary to our hypothesis, more Instagram usage was associated with lower perceived extremity of upward social comparisons. This model explained 8% (*p* < 0.01) of the variance of physical self-esteem and was not improved by the addition of moderators or covariates.

##### 3.2.2.3 Academic self-esteem

In the academic self-esteem models (Figures 2, 3), Instagram use was not directly or indirectly associated with academic self-esteem when considering perceived exposure as the mediator. However, contrary to our hypothesis, we found a partial positive mediation between Instagram use and academic self-esteem. More specifically, as mentioned before, higher usage of Instagram was negatively related to perceived extremity of upward social comparisons which was contrary to our hypothesis. Perceived exposure to upward social comparisons was negatively associated with academic self-esteem. This significative relation was unexpected. Finally, when controlling for the mediators, Instagram usage was negatively associated with academic self-esteem (*c’* = −0.06, *t* = −2.00, *p* = 0.05). This model explained 8% (*p* < 0.01) of the variance in academic self-esteem, and results remained unchanged with the addition of moderators or covariates.

#### 3.2.3 Social media platform

**Figure 4:**
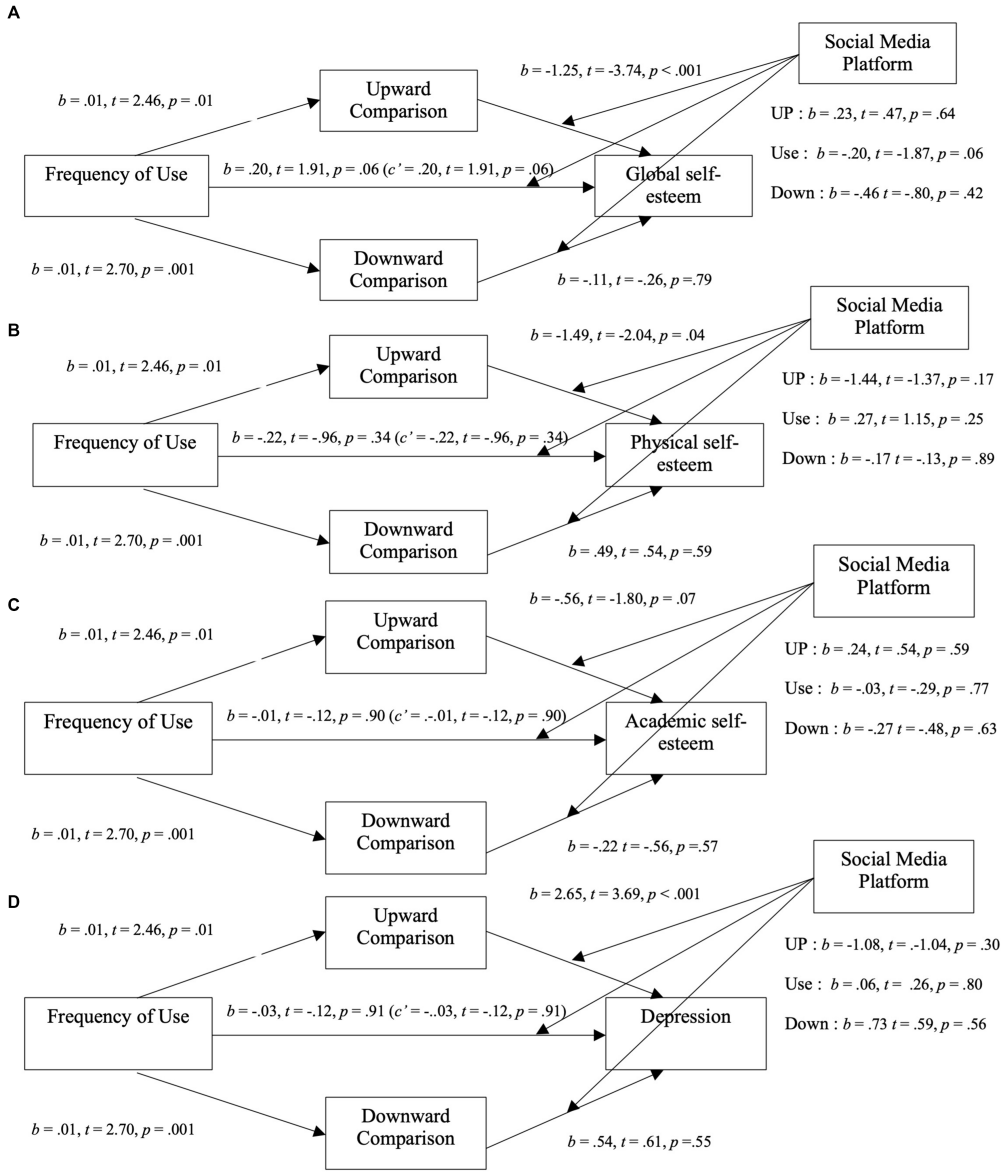
Moderated mediation analysis for mental health variables through the exposure to social comparison with social media platform as a moderator. **(A)** Moderated mediation analysis between the frequency of SNSs use and global self-esteem through the exposure to social comparison (upward and downward) with social media platform as a moderator. **(B)** Moderated mediation analysis between the frequency of SNSs use and physical self-esteem through the exposure to social comparison (upward and downward) with social media platform as a moderator. **(C)** Moderated mediation analysis between the frequency of SNSs use and academic self-esteem through the exposure to social comparison (upward and downward) with social media platform as a moderator. **(D)** Moderated mediation analysis between the frequency of SNSs use and depression through the exposure to social comparison (upward and downward) with social media platform as a moderator.

**Figure 5:**
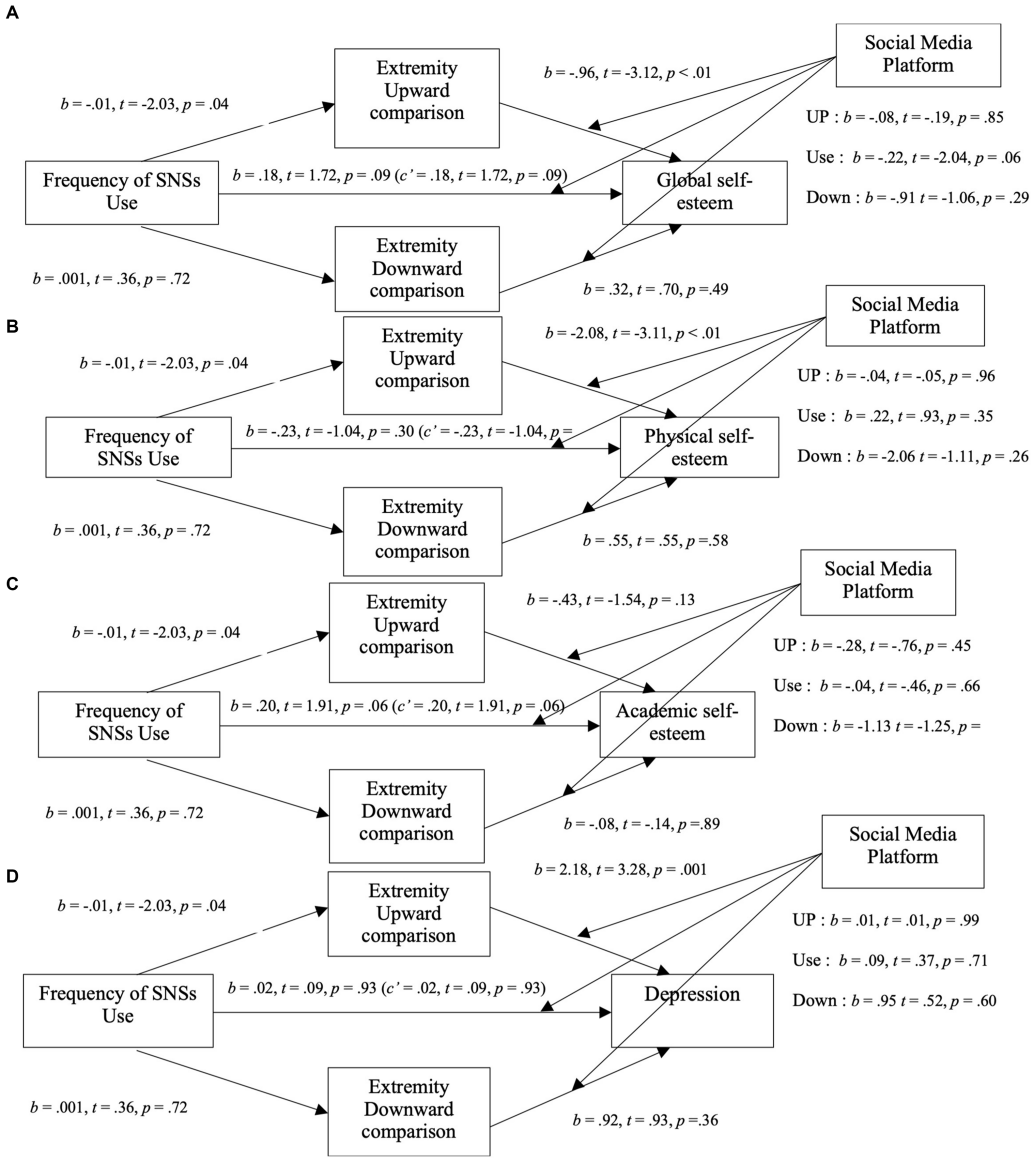
Moderated mediation analysis for mental health variables through the extremity of social comparison with social media platform as a moderator. **(A)** Moderated mediation analysis between the frequency of SNSs use and global self-esteem through the extremity of social comparison (upward and downward) with social media platform as a moderator. **(B)** Moderated mediation analysis between the frequency of SNSs use and physical self-esteem through the extremity of social comparison (upward and downward) with social media platform as a moderator. **(C)** Moderated mediation analysis between the frequency of SNSs use and academic self-esteem through the extremity of social comparison (upward and downward) with social media platform as a moderator. **(D)** Moderated mediation analysis between the frequency of SNSs use and depression through the extremity of social comparison (upward and downward) with social media platform as a moderator.

Moderated mediation models looking at the relation between SNSs usage and our dependent variables with perceived exposure to and extremity of social comparisons as mediators were tested with the addition of social media as a moderator (Facebook or Instagram) on the direct link and between the mediators and our DVs (Figures 4, 5). SNSs usage relation with global self-esteem and physical self-esteem were fully mediated by perceived exposure and by perceived extremity to upward comparisons. Similarly to our previous analysis on specific SNSs, this mediation included a positive relation between SNSs usage and perceived exposure to upward social comparisons and a negative relation between SNs usage and perceived extremity of social comparisons. The models on academic self-esteem showed no direct or indirect relation of SNSs usage on this type of self-esteem. Crucially, no significant interactions were found suggesting that, overall, these relations are not modulated by the type of social media platform.

#### 3.2.4 Exploratory—depression

**Figure 6:**
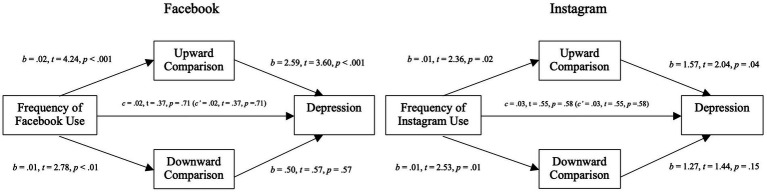
Mediation analysis for self-esteem variables through the exposure to social comparison in Study 2. Left: Mediation analysis between the frequency of Facebook use and depression through the exposure to social comparison (upward and downward). Right: Mediation analysis between the frequency of Instagram use and depression through the exposure to social comparison (upward and downward).

**Figure 7:**
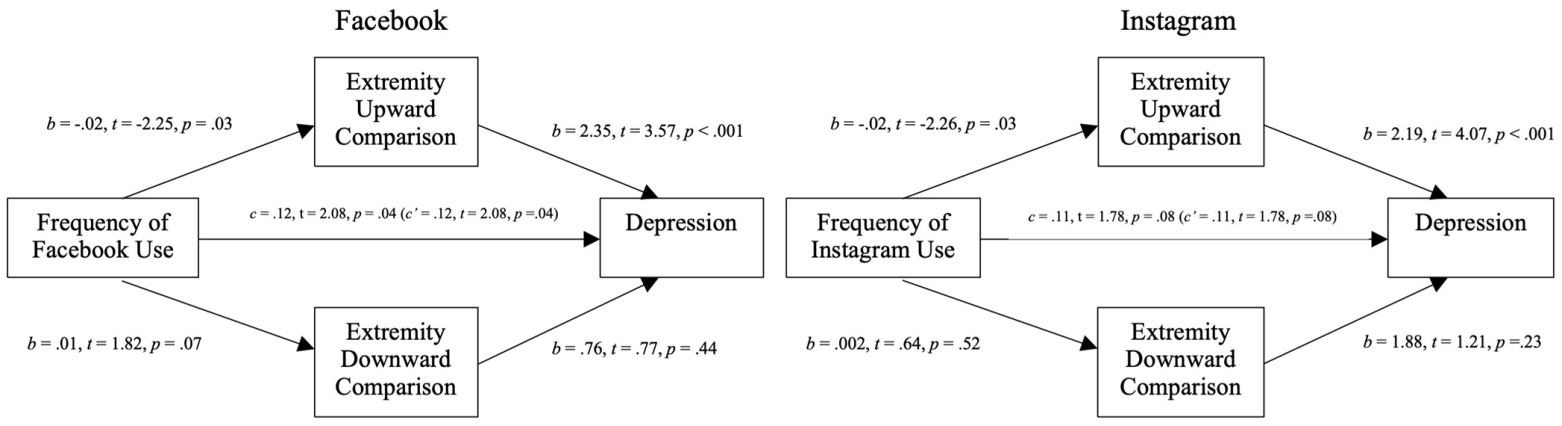
Mediation analysis for self-esteem variables through extremity of social comparison in Study 2. Left: Mediation analysis between the frequency of Facebook use and depression through the extremity of social comparison (upward and downward). Right: Mediation analysis between the frequency of Instagram use and depression through the extremity of social comparison (upward and downward).

In addition to the analysis presented in the preregistration, we investigated the mediating effect of perceived exposure to (Figure 6) and extremity of (Figure 7) social comparisons on Facebook (left side) and Instagram (right side) on users’ depressive symptoms.

##### 3.2.4.1 Facebook condition

The relation between usage and depressive symptoms was fully mediated by perceived exposure to upward social comparisons (*b* = 0.05; 95% CI [0.02, 0.11]), where higher usage was associated with more perceived exposure to upward social comparisons which was related to higher depressive symptoms (*b* = 2.59, *t* = 3.60, *p* < 0.001). The models explained 9% (*p* < 0.01) of depression variance with no improvement from additional moderators.

Contrary to our hypothesis, we found a partially mediated negative relation between Facebook usage and depressive symptoms with extremity of social comparisons as the mediators (*b* = −0.05; 95% CI [−0.07, −0.01]). More specifically, while we found that perceived extremity of upward social comparisons was positively related with depression (*b* = 2.35, *t* = 3.57, *p* < 0.001), as mentioned before, more Facebook usage was associated with lower perceived extremity of upward social comparisons. When controlling for mediators, higher Facebook usage was positively related to depressive symptoms (*c’* = 0.12, *t =* 2.08, *p* = 0.04). This model explained 7% (*p* < 0.01) of the variance in depression with no improvement from additional moderators.

##### 3.2.4.2 Instagram condition

The relation between usage and depressive symptoms was fully mediated by perceived exposure to upward social comparisons (*b* = 0.2; 95% CI [0.001, 0.06]), where higher usage was associated with more perceived exposure to upward social comparisons which was related to higher depressive symptoms (*b* = 1.57, *t* = 2.04, *p* = 0.04). The model explained 5% (*p* = 0.02) of depression variance with no improvement from additional moderators.

Contrary to our hypothesis, we found fully mediated negative relation between Instagram usage and depressive symptoms with perceived extremity of social comparisons as the mediators (*b* = −0.04; 95% CI [−0.08, −0.01]). More specifically, while we found that perceived extremity of upward social comparisons was positively related with depression (*b* = 2.19, *t* = 4.07, *p* < 0.001), more Instagram usage was surprisingly associated with lower perceived extremity of upward social comparisons. This model explained 8% (*p* < 0.01) of the variance in depression with no improvement from additional moderators.

Moderated mediation models with the type of SNS as a moderator showed no significant interaction effect (see Figure 4).

### 3.3 Discussion

Compared to study 1, study 2 adopted a more comprehensive approach by (1) examining both the perceived exposure to and extremity of social comparisons, and (2) using a new measure of physical self-esteem and adding a control dependent variable (academic self-esteem) and (3) testing potential moderators: social feedback—specifically the quantity and valence of feedback—and gender. Moreover, we explored the effects of SNSs usage and social comparisons on depressive symptoms and tested whether our effects were modulated by the platform (Facebook vs. Instagram).

Specifically, for Facebook, our hypotheses regarding mediating role of social comparisons were partially confirmed. The findings showed that the negative relationship between Facebook use and global self-esteem is fully mediated by the exposure to upward comparisons. The more participants reported to use Facebook, the more the perceived to be exposed to upward comparisons which was in turn associated with them reporting lower global self-esteem. This result is consistent with previous research (Midgley et al., 2021; Vogel et al., 2014). Similarly, the relationship between Facebook use and physical self-esteem is fully mediated by perceived exposure to upward social comparisons on Facebook. This result is consistent with previous research as well (Scully et al., 2020; Prieler et al., 2021). Additionally, when the quantity of social feedback was introduced as a moderator, the model demonstrated an even stronger fit. Participants who frequently used Facebook and received a large amount of social feedback had better physical self-esteem which is in line with Wang et al. (2020), who explored the mediating role of positive social feedback in the relationship between SNSs use and physical self-esteem (Wang et al., 2020). While Wang et al. considered positive social feedback as a mediator, our results showed a moderating effect of the quantity of social feedback (irrespective of the valence), providing alternative perspectives on the influence of social feedback on physical self-esteem in the context of SNSs use. Regarding the perceived extremity of social comparisons, surprising results were found. In line with our hypothesis, we did find that the more extreme participants perceived their upward social comparisons (i.e., greater distance between the profiles and their own social status), the lower their global and physical self-esteem were which aligns with the findings of Midgley et al. (2021). However, contrary to our hypothesis, more active users tended to make less extreme upward comparisons, which reduced the negative relation between the perceived extremity of upward social comparisons and self-esteem (global and physical).

Regarding Instagram, our hypotheses relative to the exposure to and extremity of social comparisons on Instagram were also partially confirmed. As hypothesized, the relationship between Instagram use and both overall and physical self-esteem is mediated by the exposure to upward social comparisons on Instagram—where usage is linked to a higher exposure to upward social comparisons which in turn is associated with lower global and physical self-esteem. These results are consistent with previous research (Vogel et al., 2014; Midgley et al., 2021). Regarding the extremity of social comparisons, similar surprising results to those found on Facebook were observed for Instagram. Specifically, the relationship between Instagram use and both overall and physical self-esteem was also positively mediated by the extremity of upward comparisons where more active users tended to make less extreme upward comparisons resulting in a better self-esteem (global and physical). However, the same negative association was found between the extremity of upward social comparisons and self-esteem, suggesting that participants who made the more extreme upward social comparisons on Instagram (greater distance between the comparison target and the individual) had lower global and physical self-esteem, which is consistent with the findings of Midgley et al. (2021). Lastly, our hypotheses regarding academic self-esteem were confirmed for both Facebook and Instagram. Neither the exposure to nor the extremity of social comparisons mediated the relationship between SNSs use (Facebook and Instagram) and academic self-esteem. These findings suggest that while upward social comparisons on SNSs may negatively impact self-esteem, the focus of these comparisons may determine which dimension of self-esteem are affected.

On an exploratory basis, we also investigated the effects of SNSs use and social comparisons on depression, as several studies have highlighted positive associations between SNSs use and depression (Liu and Brown, 2014; Yoon et al., 2019). On Facebook, the exposure to upward social comparisons mediated the relationship between frequency of use and depressive symptoms, where the more participants engaged in upward comparisons, the more depressive symptoms they reported. For the extremity, a surprising negative mediation path was found indicating that more active users made less extreme upward comparisons resulting in less depressive symptoms. However, both the exposure to and the extremity of upward comparisons were positive predictors of higher depressive symptoms, which is consistent with previous research (Liu and Brown, 2014; Lup et al., 2015; Yoon et al., 2019). We found similar results for Instagram.

Finally, when considering the effects of SNSs usage on academic self-esteem, results showed that for most models there was no direct or indirect relation of SNSs usage on this dimension of self-esteem (except for the model of Instagram use with the extremity of upward social comparison). This can be taken as evidence that the impacts of SNS usage through upward social comparison on self-esteem are somewhat specific to dimensions related to the content presented in the social media platform.

As mentioned before, a negative relationship was observed between the frequency of SNSs use and the extremity of upward comparisons, which is inconsistent with much of the existing literature. While previous studies have suggested a positive or neutral relationship between usage and the extremity of comparisons (Vogel et al., 2014; Midgley et al., 2021) our findings suggest that frequent SNSs users engage in less extreme upward comparisons. One potential explanation is habituation—frequent users may become desensitized to idealized content over time, perceiving it as more typical or attainable and thus rating comparisons as less extreme. Another possibility is self-selection bias—frequent users may curate their feeds more selectively over time, to include more relatable or inspirational content and unfollow accounts that trigger negative affect, leading to less extreme comparison with the profiles they follow. At the same time, it is important to consider measurement-related limitations. The scale used to assess extremity of upward comparisons relies on participants’ subjective perception of how different others on SNSs are from themselves. However, these judgments may be influenced by social desirability, personal standards, or familiarity with platform norms. It is also possible that the measure did not fully capture the nuanced affective or aspirational dimensions of what makes a comparison feel “extreme.”

However, a more important finding is the negative relationship between the extremity of social comparisons and self-esteem, which aligns with previous research. Extreme upward comparisons—where there is a large gap between the self and the comparison target—are associated with lower self-esteem (Midgley et al., 2021). This suggests that, regardless of how frequently individuals use SNSs, engaging in extreme comparisons can have a detrimental effect on self-esteem, supporting the broader social comparisons literature.

When we included SNSs (Facebook vs. Instagram) as a moderator in our models, no significant interactions effects were found (*p* > 0.05), indicating that overall, the results for both platforms were similar and did not differ significantly—contradicting our initial hypothesis which proposed that Instagram use would have stronger negative effects on self-esteem due to its image-based nature. This suggests that the influence of SNSs on self-esteem and social comparisons may be more consistent across platforms than initially expected. One explanation could be that SNSs platforms are becoming increasingly alike, particularly since Meta’s consolidation of features across its platforms. For instance, features such as Reels and Stories, once unique to Instagram, are now available on Facebook, which may reduce distinctions in user experience and psychological outcomes across platforms (VanDyke, 2020).

When social feedback was introduced as a moderator, no significant interactions were observed, except in the case of physical self-esteem on Facebook. Specifically, an interaction was identified between the frequency of Facebook use and the quantity of social feedback received on the platform, contradicting our initial hypothesis and prior findings in the literature (Limniou et al., 2022). This interaction revealed a small positive effect on physical self-esteem, such that users who were more active on Facebook and received greater amounts of social feedback reported higher physical self-esteem. This significant interaction for Facebook could be explained by the proximity of Facebook “friends” compared to Instagram followers. Indeed, most Facebook friends are people closer to the user, such as friends or family, who tend to interact more with the user’s content by liking posts or leaving positive comments (Limniou et al., 2022). In contrast, on Instagram, followers often consist of more distant or unfamiliar people, who are less likely to engage with the content posted (Limniou et al., 2022). This difference in the closeness of the people on the platforms could explain why no significant link with social feedback was found for Instagram. This suggests that social feedback can have a somewhat protective role on physical self-esteem. Additionally, when gender was included as a mediator in our models, no significant interaction was found, which contrasts with previous findings in the literature (Barthorpe et al., 2020; Kelly et al., 2019; Twenge et al., 2022). This lack of interaction may be attributed to the growing presence of men on social media and the rise of trends such as “fitspiration,” which have increased opportunities for upward comparisons among male users (Tiggemann and Anderberg, 2020).

Overall, these results partially align with our hypotheses. However, one finding is consistent for all our results: upward comparisons—both in terms of exposure and extremity—is a negative predictor of self-esteem (global and physical) and a positive predictor of depressive symptoms strongly suggesting that upward social comparisons is a key mechanism in the complex relationships between social media and its consequences. Moderate levels of upward comparisons, involving individuals slightly above one’s own standing (Diel et al., 2021) and balanced with a similar number of downward comparisons, are unlikely to be particularly harmful. After all, social comparisons did not emerge with SNSs; it is a phylogenetically ancient ability that has likely been instrumental to navigating social hierarchies throughout human evolutionary history (Benítez and Brosnan, 2020; Gilbert et al., 1995). According to ranking theory, social comparisons is a mechanism through which individuals gather information about their position in the social hierarchy (Gilbert, 2001). This process plays a crucial role in shaping self-evaluation and mood, as individuals adjust their behavior based on their perceived social rank (Gilbert, 1992). Throughout evolution, when individuals perceived themselves as having a low rank, it was evolutionarily adaptive to adopt what is known as a damage limitation strategy (Gilbert, 1992). This strategy involves displaying more submissive behavior, lowering self-confidence, reducing social exploration, and using nonverbal cues of submission, such as gaze avoidance (Gilbert, 2001). This theoretical framework helps explain why upward social comparisons are often linked to diminished self-esteem and mood.

## 4 General discussion

Early research on the effects of SNSs predominantly focused on the linear relationship between SNSs use and wellbeing, often neglecting the complex nature of their interaction (Kross et al., 2021). While there has been a growing effort to elucidate mechanisms linking SNSs use to mental health (Meier and Johnson, 2022; Verduyn et al., 2020), this endeavor remains in its early stages. A key gap in current knowledge is our limited mechanistic insight into how SNSs impact us (Kross et al., 2021).

The aim of these two studies was to gain a deeper understanding of the mechanisms mediating the relationship between SNSs use and self-esteem, with a particular focus on social comparisons, which we believe is central to these effects. More specifically, our results highlighted two key aspects of upward social comparisons—exposure and extremity—as critical factors linking SNSs use to affective outcomes such as self-esteem and depression. Indeed, the perceived exposure to upward comparisons negatively mediated the relationship between SNSs use and self-esteem, as well as the relationship between SNSs use and depressive symptoms. However, the mediation path was positive for extremity, suggesting that SNSs usage is associated to less extreme upward social comparisons which reduces the direct negative link found between the extremity of upward comparisons and affective outcomes. The relations between upward social comparisons—both in terms of exposure and extremity—and detrimental effects on self-esteem and emotional wellbeing were the most consistent of our results. These findings underscore the critical impact of various dimensions of upward social comparisons on SNSs, highlighting their role in the decline of self-esteem and the increase in depressive symptoms. Both the exposure to upward comparisons and their extreme nature are central contributors to the emotional consequences of social media use (Gerber et al., 2018; Schmuck et al., 2019; Irmer and Schmiedek, 2023). These results support previous research suggesting that frequent engagement in upward comparisons may exacerbate negative emotional outcomes, including lower self-esteem and increased depressive symptoms, particularly when the comparisons are extreme (Midgley et al., 2021).

These results are in line with predictions from ranking theory (Gilbert, 1992) and the existing literature (Gerber et al., 2018; Schmuck et al., 2019; Irmer and Schmiedek, 2023), which both suggest that the behavioral and affective consequence of upward social comparisons, which were adaptative during most of our time as a species, can in some contexts be responsible for various negative emotional consequences, including reduced self-esteem and the development of depressive symptoms. Indeed, our findings are in line with the mismatch hypothesis (Lim and Tan, 2024), which suggests that SNSs hijack our evolved social comparisons mechanisms by offering (too) abundant opportunities for upward comparisons. This hypothesis argues that SNSs inundate users with idealized content, which can lead to negative emotional outcomes.

The results from study 1, which revealed a positive relationship between SNSs use and self-esteem, suggest that additional mechanisms may be influencing this dynamic. While this paper focuses primarily on the potential negative effects of SNSs use, there is also evidence of its positive impact, such as fostering inspiration (Meier and Schäfer, 2018) and enhancing perceptions of social support through increased self-disclosure (Huang, 2016). Other positive psychological mechanisms may buffer the negative effects of social comparisons on SNSs. For instance, engaging with content that promotes self-improvement or motivation, such as fitness goals, may encourage upward comparisons that are aspirational rather than harmful, especially when users perceive the content as attainable. Social media can also serve as a space for positive identity reinforcement—for example, sharing personal milestones, achievements, or cultural identity can enhance self-concept clarity and pride being (Manago et al., 2012; Nabi et al., 2013). Furthermore, receiving positive feedback or peer validation through likes and supportive comments may enhance social capital, which is known to strengthen self-esteem and wellbeing (Boyd and Ellison, 2007). Personality traits also likely play a role in how users interpret and respond to social comparisons. For instance, individuals with high self-compassion or emotional regulation skills may be better equipped to buffer the negative impact of upward comparisons by reframing them or reducing self-criticism (Neff and Tóth-Király, 2022). Future research should examine how these protective factors interact with social comparison processes to better understand why some individuals benefit from SNS use while others are negatively affected. Ultimately, recognizing this complexity does not diminish the potential harm of excessive upward comparisons, but rather highlights the importance of understanding the nuanced interplay between social media use, individual differences, and psychological outcomes.

In both studies, when we added gender as a mediator in our models, no significant interaction effects were observed, indicating that the associations between social media use, social comparisons, and self-esteem did not differ significantly between men and women. This finding contradicts some previous research that has reported stronger negative effects of social comparison for women (Barthorpe et al., 2020; Kelly et al., 2019; Twenge et al., 2022). However, several factors may help explain this discrepancy. First, most earlier studies on social media and body image focused primarily on women, often excluding men altogether or underrepresenting them. In contrast, our sample included a more balanced gender representation, reflecting current trends showing increased male engagement with image-based platforms like Instagram. Second, shifts in digital culture may be narrowing the gender gap in social comparison experiences. With the rise of trends such as “fitspiration” and content showcasing muscular male bodies (Tiggemann and Anderberg, 2020), men are increasingly subject to idealized body standards, which may lead to similar psychological outcomes as observed in women. Indeed, recent research has found that men exposed to upward comparison content also report lower self-esteem (Fatt et al., 2019; Tiggemann and Anderberg, 2020; Barron et al., 2021), supporting the idea that men are not immune to appearance-based pressures online, which aligns with our findings. Finally, it is possible that the self-esteem measures used in our study did not fully capture gender-specific concerns. For instance, while the global and physical self-esteem scales offer valuable insights, they may not be sensitive to domains where men experience the most pressure (e.g., muscularity, dominance, athleticism). Future research could benefit from using gender-sensitive measures or exploring body image constructs specific to men, such as drive for muscularity or self-objectification.

One strength of our research was its timing, with study 1 conducted during the COVID-19 pandemic and study 2 taking place afterward. This allowed us to capture potential changes in the relation between user behavior and outcomes during the pandemic. For instance, in study 1, we observed a positive association between Instagram use and global self-esteem, a result not replicated in study 2 or supported by the existing literature. This discrepancy could be attributed to the increased socialization benefits provided by SNSs during the pandemic, which may have temporarily mitigated its usual negative effects. The findings from study 2, which aligned more closely with the established literature, suggest that the behaviors seen in study 1 were more situational and pandemic-related. While our findings in study 1 indicated a small positive association, it’s important to note that other studies found the relationship between SNSs use and mental health remained negative or even increased during the pandemic (Draženović et al., 2023; Haddad et al., 2021; Marciano et al., 2022). Additionally, study 1 found no significant link between Instagram use and physical self-esteem. As noted earlier, the questionnaire used in study 1 assessed participants’ satisfaction with individual body parts, which may have been less suited to our research goals. To address this, we used a different physical self-esteem questionnaire in study 2 which focusses on a more general appreciation of body appearance. This change in the measurement tool may also have contributed to the differences in results observed between the two studies regarding physical self-esteem. Another strength of this study is its comparative analysis of the effects of two different SNSs platforms, an area that has been relatively understudied. Our findings revealed that similar effects were present on both Facebook and Instagram, despite subtle differences in the nature of their content (Limniou et al., 2022). As noted earlier, these similarities may be partly attributed to the growing convergence of features between platforms like Facebook and Instagram, particularly since both are owned by Meta. However, the study also highlighted some important distinctions, such as differences in usage patterns. Young adults appear to be shifting toward Instagram as their primary SNSs, emphasizing the need for future research to focus more closely on this platform. Finally, including academic self-esteem as a control dependent variable enabled us to provide some of the first evidence that the effects of upward social comparisons on SNSs seems to be specific to the type of content generally present on these platforms.

Although this study provides valuable insights, several limitations should be noted. First, the correlational design restricts our ability to draw definitive conclusions about the causal role of upward social comparisons in shaping affective outcomes. Experimental studies would be better suited for establishing causality in these relationships. Second, the online data collection limited control over the accuracy and reliability of participants’ responses. While we implemented safeguards such as attention checks and filtering responses to open-ended questions, ensuring full data reliability in an online setting remains challenging. In-person studies or platforms that allow closer monitoring of participant engagement could help improve data quality in future research. Third, our analyses did not control for potentially important individual difference variables such as body image dissatisfaction, narcissism, or baseline mental health status, which could influence both social comparison processes and self-esteem outcomes. Including such variables in future research would allow for a more precise understanding of the unique effects of upward social comparisons. Finally, our study did not explore the potential influence of demographic or cultural diversity on social media behavior. Research suggests that individuals from different cultural backgrounds may engage with SNS platforms in distinct ways (Vasalou et al., 2010; Gordesli et al., 2024)—for example, using Instagram for self-expression versus Facebook for maintaining family ties. These patterns may influence both the frequency and content of social comparisons, as well as their emotional impact. The generalizability of our findings may therefore be limited, particularly in culturally diverse populations. Future studies should examine cross-cultural differences in social media use and comparison processes to gain a more nuanced understanding of these relationships. Exploring the effects of various SNSs platforms (TikTok, SnapChat, etc.) with diverse user bases and content types may also provide deeper insights into the mechanisms underlying these observed outcomes.

## 5 Conclusion

This study highlights perceived exposure to and extremity of upward social comparisons as key mechanisms linking SNSs use to impacts on self-esteem and depressive symptoms. Across two studies, we found that while perceived exposure to upward comparisons consistently mediated negative effects on mental health, extremity of comparisons was uniquely associated with lower self-esteem, suggesting that not all comparisons are equally harmful. Notably, our findings also showed that the frequency of SNSs use was not uniformly detrimental, and in some contexts—particularly during the COVID-19 pandemic—it was associated with higher self-esteem, possibly due to increased social connection or positive feedback. Given these findings, raising public awareness about the risks of excessive upward comparisons-particularly extreme ones—and promoting healthier and more intentional social media use is essential.

## Data Availability

The raw data supporting the conclusions of this article will be made available by the authors without undue reservation.

## Footnotes

^1^For more details on measures in place during the study, visit: https://www.cihi.ca/en/canadian-covid-19-intervention-timeline and https://www.cdc.gov/museum/timeline/covid19.html#Mid-2021.
