# Supplementary material for: The associations between social comparison on social media and young adults’ mental health
Source: Front Psychol. 2025 Aug 8;16:1597241. doi: 10.3389/fpsyg.2025.1597241 (PMC12370522; doi:10.3389/fpsyg.2025.1597241)
Supplement: Supplementary file 1 [file Table_1.DOCX]

**Table 1. Difference Between Sociodemographic groups and Dependent variables in Study 1**

|  |  |  | **SS** | **df** | **MS** | **F** | **p** | **η²** |
| --- | --- | --- | --- | --- | --- | --- | --- | --- |
| Global Self-esteem |  | Between groups | 161.60 | 5 | 32.32 | .91 | .47 | .03 |
|  | Ethnicity | Within groups | 5842.38 | 165 | 35.41 |  |  |  |
|  |  | Total | 6003.98 | 170 |  |  |  |  |
|  |  | Between groups | 87.75 | 2 | 43.87 | 1.25 | .29 | .02 |
|  | Gender | Within groups | 5916.23 | 168 | 35.22 |  |  |  |
|  |  | Total | 6003.98 | 170 |  |  |  |  |
|  |  | Between groups | 3400.53 | 5 | 680.11 | 1.28 | .28 | .04 |
| Physical Self-esteem | Ethnicity | Within groups | 87026.77 | 163 | 533.91 |  |  |  |
|  |  | Total | 90427.30 | 168 |  |  |  |  |
|  |  | Between groups | 10.17 | 1 | 10.17 | .019 | .89 | .01 |
|  | Gender | Within groups | 90417.14 | 167 | 541.42 |  |  |  |
|  |  | Total | 90427.30 | 168 |  |  |  |  |

| **Table 2. Difference Between Sociodemographic groups and Dependent variables on Facebook in Study 2** |
| --- |

|  |  |  | **SS** | **df** | **MS** | **F** | **p** | **η²** |
| --- | --- | --- | --- | --- | --- | --- | --- | --- |
| Global Self-esteem |  | Between groups | 593.38 | 5 | 118.68 | 4.42 | .005 | .08 |
|  | Ethnicity | Within groups | 6968.72 | 201 | 34.67 |  |  |  |
|  |  | Total | 7562.10 | 206 |  |  |  |  |
|  |  | Between groups | 204.75 | 5 | 40.95 | 1.12 | .35 | .03 |
|  | Education | Within groups | 7357.34 | 201 |  |  |  |  |
|  |  | Total | 75.10 | 206 |  |  |  |  |
|  |  | Between groups | 617.19 | 3 | 205.73 | 6.01 | < .001 | .08 |
|  | Gender | Within groups | 6944.91 | 203 | 34.21 |  |  |  |
|  |  | Total | 7562.10 | 206 |  |  |  |  |
|  |  | Between groups | 3940.74 | 5 | 788.15 | 4.32 | <.001 | .10 |
| Physical Self-esteem | Ethnicity | Within groups | 36680.34 | 201 | 182.49 |  |  |  |
|  |  | Total | 40621.08 | 206 |  |  |  |  |
|  |  | Between groups | 1680.61 | 5 | 336.12 | 1.735 | .13 | .04 |
|  | Education | Within groups | 38940.47 | 201 | 193.73 |  |  |  |
|  |  | Total | 40621.08 | 206 |  |  |  |  |
|  |  | Between groups | 2599.37 | 3 | 866.46 | 4.63 | .004 | .06 |
|  | Gender | Within groups | 38021.71 | 203 | 187.30 |  |  |  |
|  |  | Total | 40621.08 | 206 |  |  |  |  |
| Depression |  | Between groups | 2564.10 | 5 | 513.00 | 3.32 | .01 | .08 |
|  | Ethnicity | Within groups | 31092.61 | 201 | 154.69 |  |  |  |
|  |  | Total | 33657.60 | 206 |  |  |  |  |
|  |  | Between groups | 391.93 | 5 | 78.39 | .47 | .80 | .01 |
|  | Education | Within groups | 33265.67 | 201 | 165.50 |  |  |  |
|  |  | Total | 33657.60 | 206 |  |  |  |  |
|  |  | Between groups | 2229.91 | 3 | 743.30 | 4.80 | .003 | .07 |
|  | Gender | Within groups | 31427.70 | 203 | 154.82 |  |  |  |
|  |  | Total | 33657.60 | 206 |  |  |  |  |
| Differences were found for ethnicity (between Caucasian and African) and gender (male and female) in global self-esteem, physical self-esteem, and depression. No differences were observed for education level. | | | | | | | | |

| **Table 3. Difference Between Sociodemographic groups and Dependent variables on Instagram in Study 2** | | | | | | | | |  |
| --- | --- | --- | --- | --- | --- | --- | --- | --- | --- |
|  |  |  | **SS** | **df** | **MS** | **F** | **p** | **η²** | |
| Global Self-esteem |  | Between groups | 196.07 | 5 | 39.22 | 1.05 | .39 | .03 | |
|  | Ethnicity | Within groups | 7492.94 | 200 | 37.47 |  |  |  | |
|  |  | Total | 7689.02 | 205 |  |  |  |  | |
|  |  | Between groups | 206.11 | 5 | 41.22 | 1.10 | .36 | .03 | |
|  | Education | Within groups | 7482.91 | 200 | 37.42 |  |  |  | |
|  |  | Total | 7689.02 | 205 |  |  |  |  | |
|  |  | Between groups | 526.17 | 3 | 175.39 | 4.95 | .002 | .07 | |
|  | Gender | Within groups | 7162.84 | 202 | 35.46 |  |  |  | |
|  |  | Total | 7689.02 |  |  |  |  |  | |
|  |  | Between groups | 2052.92 | 5 | 410.59 | 3.02 | .01 | .07 | |
| Physical Self-esteem | Ethnicity | Within groups | 27163.57 | 200 | 135.82 |  |  |  | |
|  |  | Total | 29216.49 | 205 |  |  |  |  | |
|  |  | Between groups | 260.06 | 5 | 52.01 | .36 | .88 | .01 | |
|  | Education | Within groups | 28956.43 | 200 | 144.78 |  |  |  | |
|  |  | Total | 29216.49 | 205 |  |  |  |  | |
|  |  | Between groups | 3215.90 | 3 | 1071.97 | 8.33 | <.001 | .11 | |
|  | Gender | Within groups | 26000.59 | 202 | 128.72 |  |  |  | |
|  |  | Total | 29216.49 | 205 |  |  |  |  | |
| Depression |  | Between groups | 1321.56 | 5 | 264.31 | 1.54 | .18 | .04 | |
|  | Ethnicity | Within groups | 34446.81 | 200 | 172.23 |  |  |  | |
|  |  | Total | 35768.37 | 205 |  |  |  |  | |
|  |  | Between groups | 1260.11 | 5 | 252.02 | 1.46 | .20 | .04 | |
|  | Education | Within groups | 34508.27 | 200 | 172.54 |  |  |  | |
|  |  | Total | 35768.38 | 205 |  |  |  |  | |
|  |  | Between groups | 2353.59 | 3 | 784.53 | 4.74 | .003 | .07 | |
|  | Gender | Within groups | 33414.791 | 202 | 165.42 |  |  |  | |
|  |  | Total | 35768.37 | 205 |  |  |  |  | |
| Differences were found between genders (male and female) for global self-esteem, physical self-esteem, and depression. There was a difference in physical self-esteem based on ethnicity (between Caucasian and African). | | | | | | | | | |
